# Computational Insights into Excited State Intramolecular Double Proton Transfer Behavior Associated with Atomic Electronegativity for Bis(2′-benzothiazolyl)hydroquinone

**DOI:** 10.3390/molecules28165951

**Published:** 2023-08-08

**Authors:** Jinfeng Zhao, Chang Liu

**Affiliations:** College of Physical Science and Technology, Shenyang Normal University, Shenyang 110034, China; lc20021210202307@163.com

**Keywords:** excited state hydrogen bond, excited state double proton transfer, atomic electronegativity, charge reorganization, potential energy surface

## Abstract

Inspired by the distinguished regulated photochemical and photophysical properties of 2-(2′-hydroxyphenyl)benzazole derivatives, in this work, the novel bis(2′-benzothiazolyl)hydroquinone (BBTHQ) fluorophore is explored, looking at its photo-induced behaviors associated with different substituted atomic electronegativities, i.e., BBTHQ-SO, BBTHQ-SS and BBTHQ-Se compounds. From the structural changes, infrared (IR) vibrational variations and simulated core-valence bifurcation (CVB) indexes for the dual hydrogen bonds for the three BBTHQ derivatives, we see that low atomic electronegativity could be conducive to enhancing hydrogen bonding effects in the S_1_ state. Particularly, the O_4_-H_5_⋯N_6_ of BBTHQ-SO and the O_1_-H_2_⋯N_3_ of BBTHQ-SSe could be strengthened to be more intensive in the S_1_ state, respectively. Looking into the charge recombination induced by photoexcitation, we confirm a favorable ESDPT trend deriving from the charge reorganization of the dual hydrogen bonding regions. By constructing the potential energy surfaces (PESs) along with the ESDPT paths for the BBTHQ-SO, BBTHQ-SS and BBTHQ-Se compounds, we not only unveil stepwise ESDPT behaviors, but also present an atomic electronegativity-regulated ESDPT mechanism.

## 1. Introduction

As one of the most elementary chemical events, proton transfer (PT) should be ubiquitous throughout nature—which is very significant due to its reversibility [1]. In particular, excited state intramolecular proton transfer (ESIPT) is recognized as being vital in the excited-state dynamics field via its photo-induced excitation behaviors [2,3,4]. Undeniably, among the photochemical and photophysical reactions of organic compounds, ESIPT tautomerism between a proton donor and acceptor is one of the most studied and most promising photoreactions. For organic compounds with ESIPT characteristics, the most useful result after excitation may be the double fluorescence phenomenon. Short wavelength fluorescence is derived from the initial structure, while the long wavelength emission is derived from the proton-transfer tautomer structure. It has to be said that the isomer structure before and after proton transfer is in transient dynamic equilibrium in the excited state, while they own different electronic structural properties. It is because of this characteristic that ESIPT compounds exhibit different spectral behaviors in different surrounding environments. Due to the large Stokes shift (i.e., 6000–12,000 cm^−1^) [5], the easily modulated switching between these two fluorescence configurations greatly promotes the design of novel photoelectric materials, the screening of fluorescent probe materials, the preparation of single molecules in LEDs, etc. [6,7,8,9,10].

There is no denying that applications toward chemical sensing should be by far the most important aspect of ESIPT probes, which has been demonstrated in the detection of environmental molecules and biomolecules [11,12,13,14]. As we all know, multi-hydrogen bonds cannot be avoided in practical applications—especially in real environments or in vivo. Therefore, the application of chemical sensing in environments where multiple hydrogen bonding chains exist could be very complicated. Given the potential intrinsic properties in the mimicking of proton dynamics in biological systems, in recent years, the ESIPT behaviors involved in incorporating the transfer of two or more protons are of very great interest and fascinatination [15]. As the most basic and simplest molecular model of multi-proton transfer, a novel molecular system containing two intramolecular hydrogen bonds and capable of excited state intramolecular double proton transfer (ESDPT) has attracted much attention, as the clarification of the mechanism of this kind of ESDPT reaction has laid a solid foundation for people to further explore the dynamics of multi-proton transfer behaviors in biosystems [16,17,18]. Hypothetically, for ESDPT compounds, when the dual protons are able to perform PT reactions in an excited state, the fluorescence peak of the isomer structure with the double proton transfer might be larger than that of the isomer structure with the single proton transfer, which reveals an interesting feature of perspective biological labels and scintillators [19]. Particularly, these doubly active groups can, in fact, have robust features—and in some cases better overall properties and applicability—than mono ESIPT probes and other types of chemosensors. In addition, if fluorescence wavelengths can be larger than in the near-infrared (NIR) region, then such molecular materials will be more practical, since the light in the NIR range possess the characteristics of fast analysis speeds, a wide range of detection samples, high signal-to-noise ratios, and so forth. In particular, for the applications of NIR light, sample pretreatment is simple or they require no pretreatment, which cannot damage the sample that is to be tested and also cannot pollute the surrounding environment. Thus, it is necessary to elaborate and regulate relative ESDPT behaviors reasonably.

**Scheme 1:**
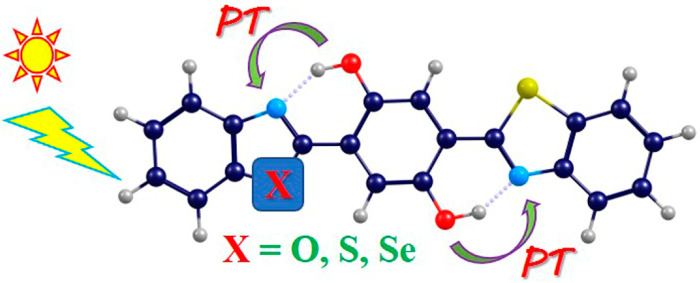
Mechanism of atomic electronegativity-associated ESPT for BBTHQ derivatives.

**Figure 1:**
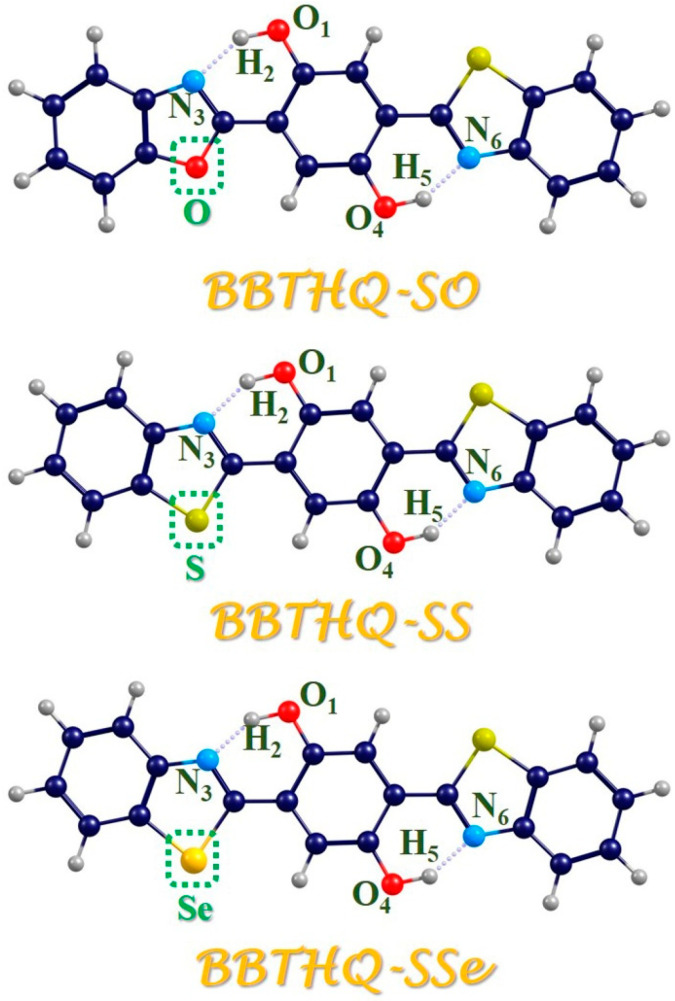
Structures of BBTHQ-SO, BBTHQ-SS and BBTHQ-Se.

As is well known, in regulating the excited state dynamic behaviors of complex molecules, the push–pull electronic groups are usually substituted to change the surrounding environments (i.e., solvent effects or pH values), to extend π-conjugation structures and so forth [20,21,22,23,24]. Nevertheless, it is relatively uncommon to regulate the excited state process by changing the atomic electronegativity. There is no denying that fluorescent molecular probes containing oxygen group elements have been shown to be very significant in the field of detection for biologically important analytes [25,26,27]. For fluorophores with ESIPT reaction characteristic, Meng and coworkers explored the sulfur-substituent 3-hydroxythioflavone experimentally [28], which revealed the occurrence of ESIPT and that the large redshift of the proton-transfer tautomer could be largely affected by the low atomic electronegativity of the sulfur atom compared with the oxygen atom. Moreover, Sun and colleagues clarified the tunable ESIPT behavior and the related antioxidant activities of 3-hydroxyflavone derivatives from a computational point of view [29]. Cao and coworkers also reported a computational investigation of the atomic electronegativity-regulated ESIPT reaction and antioxidant activity of the myricetin fluorophore [30]. Even though the classical work by Lee and coworkers reported the reactional activity of organic compounds having a certain correlation with atomic electronegativity [31], until now, there have been limited reports on atomic electronegativity being associated with ESDPT reactions experimentally and theoretically. Kungwan and coworkers reported a computational investigation on the excited state mechanism and dynamical behaviors of the typical bis(2′-benzothiazolyl)hydroquinone (BBTHQ) compound, which showed the ESDPT process in the S_1_ state [32]. In fact, only the stepwise ESDPT path was considered in Ref. [32]; the possibility of synergistic dual PT behavior was neglected. Inspired by the potential atomic electronegativity-regulated ESDPT behaviors and the significance of the overall dynamics of BBTHQ itself, in this work, we take BBTHQ as the target molecule for probing into the effects of the atomic electronegativity of oxygen group elements on ESDPT behavior in detail. In this work, we mainly focus on exploring and clarifying ESDPT behaviors associated with the different atomic electronegativities of oxygen group elements (O, S and Se) stemming from photoexcitation, as shown in Scheme 1. It is undeniable that the molecular structure of BBTHQ itself possesses symmetry, so to avoid symmetrical excited-state behavior caused by this symmetry, we only investigate the structures after unilateral replacement by the O, S and Se elements. As shown in Figure 1, the BBTHQ-SO, BBTHQ-SS (i.e., BBTHQ itself), and BBTHQ-SSe are present. Using density functional theory (DFT) and time-dependent DFT (TD-DFT) methods, we clarify the presence of photo-induced behaviors and an ESDPT mechanism associated with atomic electronegativity for the BBTHQ-SO, BBTHQ-SS, and BBTHQ-SSe compounds.

## 2. Results and Discussion

### 2.1. Photo-Induced Hydrogen Bonding Strengthening

In Figure 1, we show the main research objects of this work—i.e., BBTHQ-SO, BBTHQ-SS and BBTHQ-SSe. Meanwhile, the proton-transfer tautomers of these three compounds are shown in Figure S1, ESI^†^ (Supplementary Materials). Firstly, before making a comprehensive study, it was necessary to examine the choice of functional, because a reasonable choice of functional can truly reveal the full picture of the excited state dynamics. To vividly describe these dynamics and for comparison with the experimental absorption peak of BBTHD-SS [32], the most common different functionals were adopted to probe the vertical excitation features (listed in Table S1, ESI^†^, Supplementary Materials)—including PBE0 [33], Cam-B3LYP [34], M062X [35], mPW1PW91 [36] and ωB97XD [37]. The closest to the experimental value (440 nm) was 446 nm with the B3LYP, which confirms the rationality and correctness of adopting the B3LYP functional to explore BBTHQ and its derivatives in this work. 

It is well known that the variations of hydrogen bonding structural parameters before and after photoexcitation can reflect the excited state activity of molecules [38]; therefore, we first investigated the structural changes in the intramolecular double hydrogen bonds in the BBTHQ-SO, BBTHQ-SS and BBTHQ-SSe compounds between the S_0_ and S_1_ states. In Table 1, we present the optimized molecular structural parameters related to the dual intramolecular hydrogen bonds (O_1_-H_2_⋯N_3_ and O_4_-H_5_⋯N_6_) in both the S_0_ and S_1_ states for BBTHQ-SO, BBTHQ-SS and BBTHQ-SSe. Similarly, the structural parameters of the optimized PT tautomers involved in BBTHQ-SO, BBTHQ-SS and BBTHQ-SSe are listed in Tables S2–S4, ESI^†^ (Supplementary Materials). Compared with the case of the S_0_ state, although it can be seen that the dual hydroxy lengths were elongated and that the distances of the hydrogen bonds H_2_⋯N_3_ and H_5_⋯N_6_ were shortened with the augmentation of the bond angles (Δ(O_1_-H_2_⋯N_3_) and Δ(O_4_-H_5_⋯N_6_)) in the S_1_ state, it is worth noting that the degree of the structural changes in the BBTHQ-SO, BBTHQ-SS and BBTHQ-SSe should be different. In fact, it could be clearly seen that the variables of the H_2_⋯N_3_ lengths were 0.11849 Å, 0.13823 Å and 0.14262 Å for the BBTHQ-SO, BBTHQ-SS and BBTHQ-SSe fluorophores going from the S_0_ to the S_1_ state—while for the H_5_⋯N_6_ distances, in the same time, the variables were 0.14178 Å, 0.13823 Å and 0.13614 Å. This indicates that although photoinduced excitation can increase the strength of hydrogen bonds in the excited state [38], for BBTHQ-SO, the strengthening of the O_4_-H_5_⋯N_6_ increased significantly—while for BBTHQ-SSe, the strengthening of the O_1_-H_2_⋯N_3_ was more obvious. 

**Figure 2:**
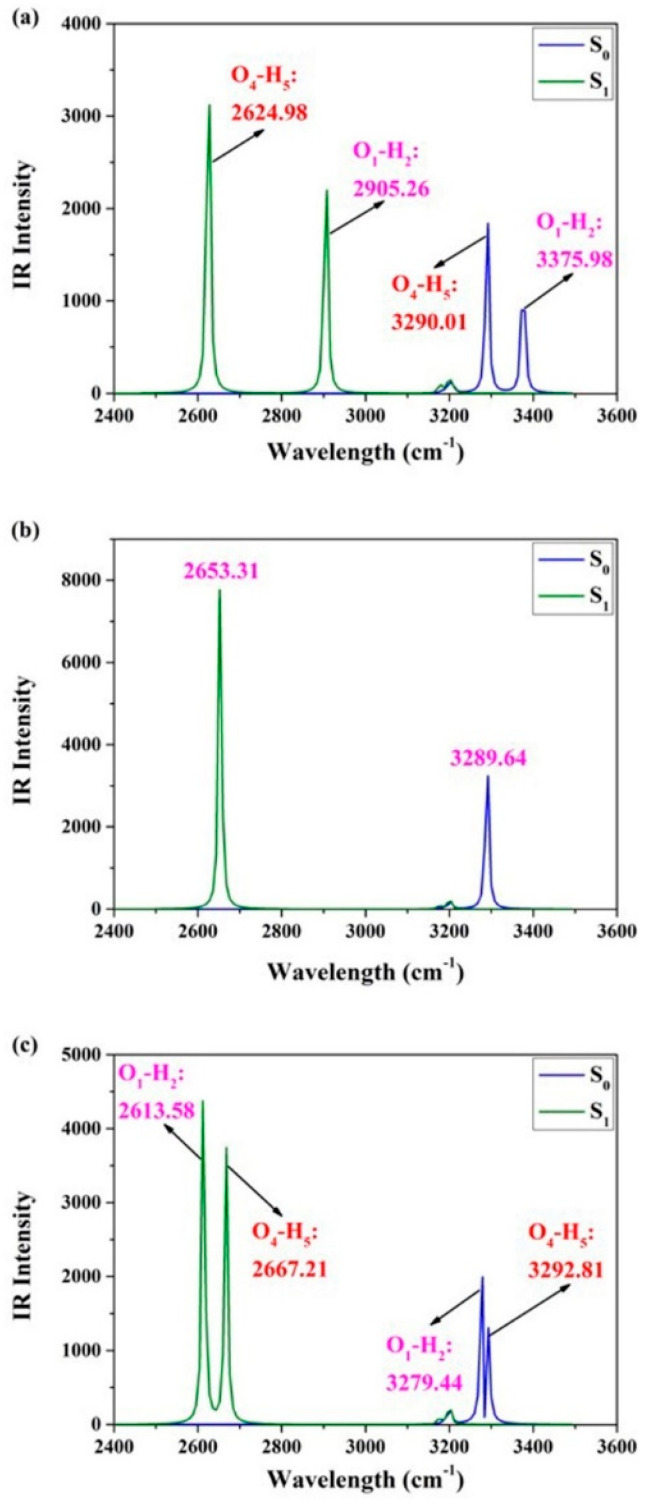
Simulated IR vibrational spectra of the O_1_-H_2_ and O_4_-H_5_ stretching vibrational mode for BBTHQ-SO (**a**), BBTHQ-SS (**b**) and BBTHQ-Se (**c**) in the S_0_ and S_1_ states.

In order to further qualitatively reveal the changes in the hydrogen bonding interactions after photoexcitation, we further investigated the infrared (IR) stretching vibration behavior of the hydroxyl moieties (O_1_-H_2_ and O_4_-H_5_) of the double hydrogen bonds. Considering the practicability and correctness of the IR vibration spectrum for clarifying the strength of the chemical bonds between the ground state and the excited states [39,40,41,42,43,44,45], the IR results of the BBTHQ-SO, BBTHQ-SS and BBTHQ-SSe are shown in Figure 2. Obviously, the stretching IR results of the hydroxyl group all showed a redshift in the S_1_ state, which reveals that photoexcitation can indeed cause excited-state hydrogen bonding interactions to become stronger. It is noteworthy that for BBTHQ-SO, the IR of the O_4_-H_5_ had a larger redshift than that of the O_1_-H_2_—while for BBTHQ-SSe, the IR of the O_1_-H_2_ showed a larger redshift than that of the O_4_-H_5_. 

To provide intuitive information concerning hydrogen bond strength from a quantitative perspective, based on the electron localization function (ELF) [46], we also calculated the core-valence bifurcation (CVB) index of the dual hydrogen bonds O_1_-H_2_⋯N_3_ and O_4_-H_5_⋯N_6_ in the S_0_ and S_1_ states. As listed in Table 2, the present simulated CVB indexes could be obtained using Multiwfn software (Version 3.8) [47]. Generally, hydrogen bonds can be written as D-H∙∙∙A, where D is the hydrogen bond donor atom and A is the hydrogen bond acceptor atom. ELF (C-V, D) represents the value of the core value dichotomy on the D atom, ELF (DH-A) represents the value of dichotomy between V (D, H) and V (A), where V (D, H) is the valence basin composed of the D and H bonded with it and V (A) is the valence basin of the atom. According to the Multiwfn software [47], the CVB index could be expressed as follows: $\mathrm{CVB}\;\mathrm{index}\;=\;\mathrm{ELF}\left(C-V,\;D\right)-\;\mathrm{ELF}\left(\mathrm{DH}-A\right)$. In agreement with the widely accepted conclusion, CVB is generally negative when the hydrogen bond present is extremely strong and has covalent characteristics. If hydrogen bonds shows a medium strength, the CVB value will be near 0. A positive value on the CVB index indicates weaker hydrogen bonding interactions [46], and the more negative the CVB index, the stronger the hydrogen bond [47]. From Table 2, it could be clearly seen that the dual hydrogen bonds O_1_-H_2_⋯N_3_ and O_4_-H_5_⋯N_6_ of the BBTHQ-SO, BBTHQ-SS and BBTHQ-SSe belonged to strong hydrogen bonding interactions in both the S_0_ and S_1_ states. Furthermore, the CVB index of the S_1_ state was more negative than that of the S_0_ state, indicating that the dual hydrogen bonds O_1_-H_2_⋯N_3_ and O_4_-H_5_⋯N_6_ were enhanced in the S_1_ state. The most remarkable thing is that the S_1_-state CVB value of the O_4_-H_5_⋯N_6_ was more negative than that of the O_1_-H_2_⋯N_3_ one for the BBTHQ-SO; however, the S_1_-state CVB value of the O_1_-H_2_⋯N_3_ was more negative than that of the O_4_-H_5_⋯N_6_ for the BBTHQ-SSe. This result could again confirm the conclusion mentioned above that the O_4_-H_5_⋯N_6_ of BBTHQ-SO and the O_1_-H_2_⋯N_3_ of BBTHQ-SSe should be further enhanced following photoexcitation.

### 2.2. Vertical Excitation

For further probing into the atomic electronegativity-associated effects on the photophysical properties for the BBTHQ-SO, BBTHQ-SS and BBTHQ-SSe compounds, here, we mainly focused on simulating the vertical excitation behavior in a hexane solvent. The transition properties of the absorption behaviors of the BBTHQ-SO, BBTHQ-SS and BBTHQ-SSe are listed in Table S5, ESI^†^ (Supplementary Materials). It could be seen that the maximum absorption peaks of these three compounds were located at 433.30 nm, 446.18 nm and 450.46 nm, respectively. This indicates that the maximum absorption peak should be redshifted with decreases in atomic electronegativity according to the order (O → S → Se). 

**Figure 3:**
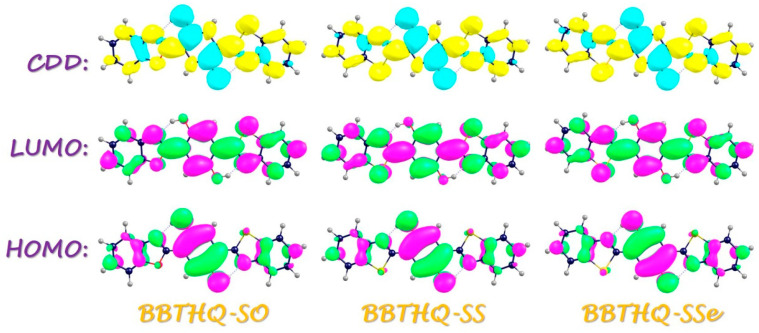
View of the HOMO and LUMO orbitals for the BBTHQ-SO, BBTHQ-SS and BBTHQ-Se fluorophores. Here, the relative CDD results are also shown.

Moreover, to further uncover the charge redistribution behavior resulting from photoexcitation [48,49,50,51,52,53,54,55,56], we also explored the frontier molecular orbitals (MOs) for the BBTHQ-SO, BBTHQ-SS and BBTHQ-SSe compounds. Since the S_0_ → S_1_ transition could mainly be reflected by the HOMO → LUMO transition with an orbital contribution greater than 98%, shown in Table S2, we only present the HOMO and LUMO orbitals for the BBTHQ-SO, BBTHQ-SS and BBTHQ-SSe in Figure 3. Clearly, all the HOMOs and LUMOs exhibited π and π* features, respectively. Attentively, during the HOMO → LUMO transition for the three compounds, the electronic densities over the O_1_ and O_4_ atoms decreased, whereas those over the N_3_ and N_6_ atoms increased in the meantime. To visualize this kind of intramolecular charge transfer (ICT) behavior, the charge density difference (CDD) results for the BBTHQ-SO, BBTHQ-SS and BBTHQ-SSe were considered between the S_1_ and S_0_ states, with the isovalue set to 0.005 (seen in Figure 3). The yellow moieties of the CDD indicate increased charge densities, while the blue regions represent reductions in charge densities during the S_0_ → S_1_ transition. Apparently, after photoexcitation, the driving force could be induced by the net charge densities shifting from hydroxy moieties to N_3_ and N_6_ atoms, which could facilitate the ESPT reaction for the BBTHQ-SO, BBTHQ-SS and BBTHQ-SSe.

The ESIPT phenomenon should be, essentially, the result of heavy atoms being affected by light, causing changes in their electron charge and other properties. By utilizing advanced techniques such as Mulliken’s charge analysis and NPA charge methods to investigate hydrogen bonding moieties within the target molecules, we may be able to gain a better understanding of how this entire process works. The computational Mulliken’s charge and NPA charge of the O_1_, H_2_, N_3_, O_4_, H_5_ and N_6_ atoms for the BBTHQ-SO, BBTHQ-SS and BBTHQ-SSe compounds are listed in Table 3. Comparing the results of the simulated Mulliken’s charge and the NPA charge, it is not difficult to find that the results of the two different methods are consistent in their changing behavior. Here, we can clearly see that after photoexcitation, the negative charge of the hydrogen bonding donor O atoms increased, while the negative charge of the hydrogen bonding acceptor N atoms decreased. It is worth noting that for the BBTHQ-SO, the change in the negative charge of the N_6_ atom was significantly larger than that of the N_3_ atom between the S_0_ and S_1_ states. Meanwhile, in the BBTHQ-SSe molecule, the change in the negative charge of the N_3_ atom was significantly larger than that of the N_6_ atom. This is consistent with the conclusion obtained from the above structural analysis (i.e., the O_4_-H_5_⋯N_6_ of BBTHQ-SO and the O_1_-H_2_⋯N_3_ of BBTHQ-SSe should be further enhanced via photoexcitation).

### 2.3. ESDPT Behaviors

Although the strengthened hydrogen bond and photo-induced charge recombination can reflect the occurrence of an ESPT reaction, the specific excited state dynamical process is unclear. Therefore, in this part, we mainly focus on clarifying the relevant ESPT reaction mechanism. Due to the existence of the double hydrogen bonds O_1_-H_2_⋯N_3_ and O_4_-H_5_⋯N_6_ in the BBTHQ-SO, BBTHQ-SS and BBTHQ-SSe, the ESDPT process is naturally the focus of this work. Along with dual hydrogen bonding paths, we constructed the potential energy surfaces (PESs) of both the S_0_ and S_1_ states for these three compounds using a restrictive optimization method [57,58,59,60]. To be more specific, by limiting the bond length of the O_1_-H_2_ and O_4_-H_5_ from 0.90 Å to 2.10 Å in accordance with a step size of 0.10 Å to optimize the overall structures, the PESs formed by the reaction coordinate of the O_1_-H_2_ and O_4_-H_5_ could be sufficient to illustrate the overall kinetic process involved in this work for BBTHQ-SO, BBTHQ-SS and BBTHQ-SSe in the S_0_ and S_1_ states. The S_0_-state PESs are shown in Figure S2, ESI^†^ (Supplementary Materials). In fact, with the increase of the length of the O_1_-H_2_ and O_4_-H_5_ bonds, we can clearly see that the total potential energy was constantly increasing, which indicates that BBTHQ-SO, BBTHQ-SS and BBTHQ-SSe cannot undergo a forward proton transfer reaction in the S_0_ state. However, due to photoexcitation, the three compounds exhibited significantly different dynamic behaviors in the S_1_ state compared with the S_0_ state. 

**Figure 4:**
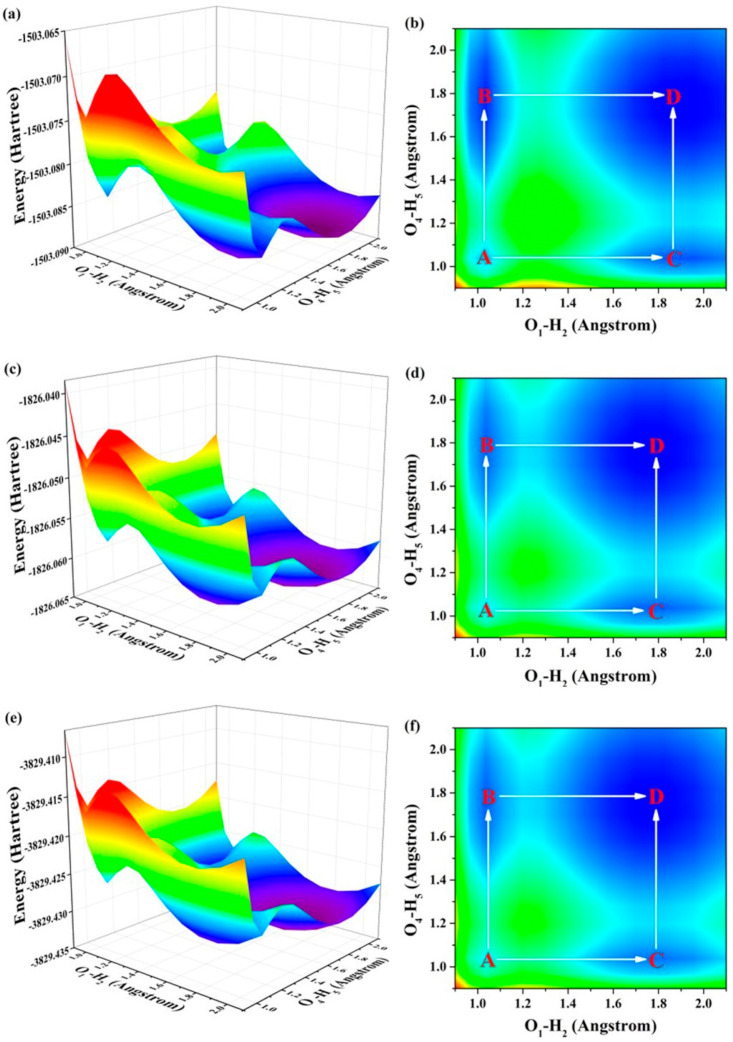
(**a**) Constructed S_1_-state PES for BBTHQ-SO; (**b**) The projective plane of the S_1_-state PES of BBTHQ-SO; (**c**) Constructed S_1_-state PES for BBTHQ-SS; (**d**) The projective plane of the S_1_-state PES of BBTHQ-SS; (**e**) Constructed S_1_-state PES for BBTHQ-SSe; (**f**) The projective plane of the S_1_-state PES of BBTHQ-SSe. The minimum points (A, B, C, and D) have been marked on the projective plane, respectively.

As shown in Figure 4, we present the S_1_-state PESs of the BBTHQ-SO, BBTHQ-SS and BBTHQ-SSe. In addition, for the sake of narration, we also present the projective plane of the S_1_-state PES in Figure 4. In the S_1_-state projective plane, we marked A, B, C and D labels to denote the optimized initial structure, the single proton-transfer form along with the hydrogen bond O_4_-H_5_⋯N_6_, the single proton-transfer tautomer along with the hydrogen bond O_1_-H_2_⋯N_3_ and the dual proton-transfer configuration for the three compounds, respectively. It is not difficult to see that the rigid symmetrical molecular structure resulted in symmetrical PESs, such as in the S_0_-state and S_1_-state BBTHQ-SS fluorophore—while for the BBTHQ-SO and BBTHQ-SSe, the PESs were not symmetrical, as the structures were non-symmetrical. From Figure 4, we can clearly see that the synergetic ESDPT behavior was inhibited, as the potential barrier of the single ESIPT path (A → B or A → C) was significantly lower than that of the synergetic ESDPT path for BBTHQ-SO, BBTHQ-SS and BBTHQ-SSe. Accordingly, the synergistic ESDPT reaction path could be excluded in this work.

In the S_1_-state PESs, our results showed that the population of the dual proton-transfer isomer increased with decreasing atomic electronegativity from O to S to Se, which revealed that the stepwise ESDPT behavior can be regulated by atomic electronegativity. To further reveal the different potential barriers of the stepwise ESDPT reaction for BBTHQ-SO, BBTHQ-SS and BBTHQ-SSe, here, we listed the potential energy barriers of the stepwise double proton transfer in Table 4. To be more specific, although it can be seen from the barrier size that stepwise ESDPT could occur along with A → C → D or A → B → D, the main ESDPT pathways of the three compounds were different. For BBTHQ-SO, compared with the 3.5574 kcal/mol of the A → C path, the A → B path with a barrier 1.7520 kcal/mol was preferred. This is consistent with the previous conclusion that the O_4_-H_5_⋯N_6_ is strengthened to be more intensive in the S_1_ state. While for BBTHQ-SSe, the opposite was true, i.e., the first ESIPT process was performed through the A → C path. For BBTHQ-SS, due to the symmetry itself, the first ESIPT reaction along with A → C or A → B should be equal. In brief, we were able to clearly unveil that BBTHQ-SO, BBTHQ-SS and BBTHQ-SSe undergo the stepwise ESDPT process. More importantly, we further present the atomic electronegativity-controlled stepwise ESDPT for BBTHQ derivatives.

## 3. Computational Methods

In this work, all simulations were performed using Gaussian 16 software [61]. DFT and TD-DFT methods were carried out to explore the S_0_-state and S_1_-state calculations based on the B3LYP functional and TZVP basis set, respectively [62,63,64,65]. In order to account for dispersion forces in all the results, the D3 version of Grimme’s dispersion was taken into account in all calculations [66,67]. Given the hexane solvent mentioned in a previous work [32], in this paper, hexane was used throughout all the simulations by using the integral equation formal variables of the polarizable continuum model (IEFPCM) model [68,69,70]. During the functional testing processes, in addition to B3LYP, we also considered the following common functionals: PBE0 [33], Cam-B3LYP [34], M062X [35], mPW1PW91 [36] and ωB97XD [37]. Based on the results of the infrared (IR) vibrational spectrum, all the optimized structures were non-virtual frequency, which successfully confirmed the stability of all the structures in this work. The photo-induced vertical excitation results were obtained based on optimized S_0_-state structures using the TD-DFT method, considering six low-lying absorption transitions, which revealed the features of the charge redistribution via frontier molecular orbitals (MOs). To interpret the ESDPT behaviors of the BBTHQ-SO, BBTHQ-SS, and BBTHQ-SSe fluorophores, we adopted the method of constructing potential energy surfaces (PESs) along with dual intramolecular hydrogen bonds (O_1_-H_2_⋯N_3_ and O_4_-H_5_⋯N_6_), based on the restrict optimization method. 

## 4. Conclusions

In summary, we mainly studied the variations in the dual hydrogen bonding behaviors and ESDPT reaction dynamics of atomic electronegativity-associated BBTHQ in hexane solvent after photoexcitation from a computational perspective. Focusing on geometrical variations and IR spectral shifts, we firstly judged the S_1_-state hydrogen bonding strengthening behavior. Based on the simulations of the CVB index, we further determined that low atomic electronegativity could contribute to hydrogen-bonding enhancement in the S_1_ state. It is worth noting for BBTHQ-SO, the strengthening of the O_4_-H_5_⋯N_6_ increased significantly, while for BBTHQ-SSe, the strengthening of the O_1_-H_2_⋯N_3_ was more obvious. Looking into the charge recombination induced by photoexcitation, we confirmed the favorable ESDPT tendency stemming from charge reorganization related to dual hydrogen bonding regions, which promote the ESDPT reaction. Then, to further explain the excited state dynamical reactions, we successfully uncovered the stepwise ESDPT mechanism by constructing the PESs for the BBTHQ-SO, BBTHQ-SS and BBTHQ-SSe compounds. Even though the stepwise ESDPT reaction was performed for three compounds, they mainly follow their own different paths. We not only elaborated the excited state dynamical behavior of the BBTHQ derivatives in detail, but also presented and unveiled the atomic electronegativity-dependent stepwise ESDPT mechanism. We sincerely wish our work may pay the way for the design and development of novel potential applications for novel 2-(2′-hydroxyphenyl)benzothiazole (HBT) derivatives.

## Figures and Tables

**Table 1:** Parameters of the bond lengths (Å) and bond angles (Δ°) involved in O_1_-H_2_⋯N_3_ and O_4_-H_5_⋯N_6_ for the BBTHQ-SO, BBTHQ-SS and BBTHQ-Se forms in the S_0_ and S_1_ states.

	BBTHQ-SO		BBTHQ-SS		BBTHQ-SSe	
	S_0_	S_1_	S_0_	S_1_	S_0_	S_1_
O_1_-H_2_	0.98488	1.01016	0.98858	1.02371	0.98914	1.02617
H_2_⋯N_3_	1.80813	1.68964	1.75430	1.61607	1.74982	1.60720
O_4_-H_5_	0.98870	1.02551	0.98858	1.02371	0.98865	1.02306
H_5_⋯N_6_	1.75141	1.60963	1.75430	1.61607	1.75384	1.61770
Δ(O_1_H_2_N_3_)	145.17	148.19	146.01	149.76	146.14	150.03
Δ(O_4_H_5_N_6_)	146.04	150.09	146.01	149.76	146.01	149.71

**Table 2:** Simulated ELF(C-V,D), ELF(DH-A) and CVB parameters related to the O_1_-H_2_⋯N_3_ and O_4_-H_5_⋯N_6_ of the BBTHQ-SO, BBTHQ-SS and BBTHQ-Se compounds in the S_0_ and S_1_ states.

		BBTHQ-SO		BBTHQ-SS		BBTHQ-SSe	
		S_0_	S_1_	S_0_	S_1_	S_0_	S_1_
O_1_-H_2_⋯N_3_	ELF(C-V,D)	0.0993	0.1010	0.1010	0.1035	0.1011	0.1038
	ELF(DH-A)	0.1592	0.2240	0.1888	0.2826	0.1932	0.2929
	CVB index	−0.0599	−0.1230	−0.0878	−0.1791	−0.0921	−0.1891
O_4_-H_5_⋯N_6_	ELF(C-V,D)	0.1010	0.1036	0.1010	0.1035	0.1010	0.1034
	ELF(DH-A)	0.1901	0.2876	0.1888	0.2826	0.1891	0.2811
	CVB index	−0.0891	−0.1840	−0.0878	−0.1791	−0.0881	−0.1777

**Table 3:** The Mulliken’s charge and NPA charge of the O_1_, H_2_, N_3_, O_4_, H_5_ and N_6_ atoms for the BBTHQ-SO, BBTHQ-SS and BBTHQ-SSe compounds in hexane solvent in both the S_0_ and S_1_ states.

		Mulliken’s Charge		NPA Charge	
	Atoms	S_0_	S_1_	S_0_	S_1_
BBTHQ-SO	O_1_	−0.282	−0.276	−0.652	−0.650
	H_2_	0.297	0.297	0.505	0.503
	N_3_	−0.148	−0.151	−0.492	−0.496
	O_4_	−0.283	−0.279	−0.653	−0.651
	H_5_	0.305	0.305	0.502	0.497
	N_6_	−0.109	−0.121	−0.471	−0.477
BBTHQ-SS	O_1_	−0.284	−0.280	−0.654	−0.652
	H_2_	0.304	0.305	0.503	0.497
	N_3_	−0.112	−0.123	−0.471	−0.478
	O_4_	−0.284	−0.280	−0.654	−0.652
	H_5_	0.304	0.305	0.503	0.497
	N_6_	−0.112	−0.123	−0.471	−0.478
BBTHQ-SSe	O_1_	−0.284	−0.280	−0.654	−0.653
	H_2_	0.305	0.305	0.503	0.497
	N_3_	−0.122	−0.135	−0.471	−0.479
	O_4_	−0.285	−0.280	−0.653	−0.652
	H_5_	0.304	0.304	0.502	0.496
	N_6_	−0.113	−0.124	−0.478	−0.485

**Table 4:** The calculated S_1_-state stepwise ESDPT potential barriers (kcal/mol) for the BBTHQ-SO, BBTHQ-SS and BBTHQ-Se compounds.

	BBTHQ-SO	BBTHQ-SS	BBTHQ-SSe
A → C	3.5574	1.8323	1.5976
C → D	1.9277	1.9971	2.0237
A → B	1.7520	1.8323	1.9976
B → D	3.8121	1.9971	1.7131

## Data Availability

Data is available on request from the corresponding author.

## Supplementary Materials

The following supporting information can be downloaded at: https://www.mdpi.com/article/10.3390/molecules28165951/s1. Figure S1: View of the proton-transfer configurations. BBTHQ-SO-PT1: single proton-transfer BBTHQ-SO along with O1-H2⋯N3; BBTHQ-SO-PT2: single proton-transfer BBTHQ-SO along with O4-H5⋯N6; BBTHQ-SO-DPT: double proton-transfer BBTHQ-SO along with O1-H2⋯N3 & O4-H5⋯N6; BBTHQ-SS-PT: single proton-transfer BBTHQ-SS along with O1-H2⋯N3 or O4-H5⋯N6; BBTHQ-SS-DPT: double proton-transfer BBTHQ-SS along with O1-H2⋯N3 & O4-H5⋯N6; BBTHQ-SSe-PT1: single proton-transfer BBTHQ-SSe along with O1-H2⋯N3; BBTHQ-SSe-PT2: single proton-transfer BBTHQ-SSe along with O4-H5⋯N6; BBTHQ-SSe-DPT: double proton-transfer BBTHQ-SSe along with O1-H2⋯N3 & O4-H5⋯N6. Figure S2: The constructed S0-sate PES for BBTHQ-SO (a), BBTHQ-SS (b) and BBTHQ-SSe (c). Table S1: Experimental and calculated maximum absorption peaks (nm) of BBTHQ in hexane solvent by TDDFT method with different functionals at TZVP basis set. Table S2: Vertical excitation energies (λ nm), oscillator strength (*f*), transition compositions and percentages for BBTHQ-SO, BBTHQ-SS and BBTHQ-Se compounds in hexane solvent. Table S3: Parameters of bond lengths (Å) and bong angles (Δ°) involved in dual hydrogen bonds for BBTHQ-SS-PT and BBTHQ-SS-DPT forms in S0 and S1 states. Table S4: Parameters of bond lengths (Å) and bong angles (Δ°) involved in dual hydrogen bonds for BBTHQ-SSe-PT1, BBTHQ-SSe-PT2 and BBTHQ-SSe-DPT forms in S0 and S1 states. Table S5: Vertical excitation energies (λ nm), oscillator strength (f), transition compositions and percentages for BBTHQ-SO, BBTHQ-SS and BBTHQ-Se compounds in hexane solvent.
