# Supplementary material for: Computational Insights into Excited State Intramolecular Double Proton Transfer Behavior Associated with Atomic Electronegativity for Bis(2′-benzothiazolyl)hydroquinone
Source: Molecules. 2023 Aug 8;28(16):5951. doi: 10.3390/molecules28165951 (PMC10458628; doi:10.3390/molecules28165951)
Supplement: Supplementary file 1 [file molecules-28-05951-s001.zip › molecules-2522224-supplementary.pdf]

Computational Insights into Excited State Intramolecular Double Proton Transfer Behavior Associated with Atomic Electronegativity for Bis(2′-benzothiazolyl)hydroquinone

Jinfeng Zhao * and Chang Liu

College of Physical Science and Technology, Shenyang Normal University, Shenyang 110034, China; lc20021210202307@163.com
* Correspondence: jfzhao1990112@163.com or jfzhao1990112@synu.edu.cn

Table S1. Experimental and calculated maximum absorption peaks (nm) of BBTHQ in hexane solvent by TDDFT method with different functionals at TZVP basis set.

PBE0	B3LYP	Cam-B3LYP	M062X	mPW1PW91	ωB97XD	Exp.[25]
431.63	446.18	393.55	384.85	432.04	390.42	440

Table S2. Parameters of bond lengths (Å) and bong angles (Δ°) involved in dual hydrogen bonds for BBTHQ-SO-PT1, BBTHQ-SO-PT2 and BBTHQ-SO-DPT forms in S_0 and S_1 states.

	BBTHQ-SO-PT1		BBTHQ-SO-PT2		BBTHQ-SO-DPT	
	S_0	S_1	S_0	S_1	S_0	S_1
O_1-H_2	1.63181	1.91396	0.98488	1.00906	0.98488	1.90354
H_2-N_3	1.05899	1.02367	1.80813	1.68592	1.80813	1.02336
O_4-H_5	0.98515	1.02502	0.98870	1.76383	0.98870	1.75253
H_5-N_6	1.76087	1.60397	1.75141	1.03633	1.75141	1.03655
$\Delta(O_1H_2N_3)$	136.94	126.07	145.17	148.21	145.17	126.57
$\Delta(O_4H_5N_6)$	146.32	150.26	146.04	134.72	146.04	135.26

Table S3. Parameters of bond lengths (Å) and bong angles (Δ°) involved in dual hydrogen bonds for BBTHQ-SS-PT and BBTHQ-SS-DPT forms in S_0 and S_1 states.

	**BBTHQ-SS-PT**		**BBTHQ-SS-DPT**	
	S_0	S_1	S_0	S_1
O_1-H_2	0.98858	1.77857	0.98858	1.77076
H_2-N_3	1.75430	1.03418	1.75430	1.03377
O_4-H_5	0.98858	1.02303	0.98858	1.77076
H_5-N_6	1.75430	1.61164	1.75430	1.03377
$\Delta(O_1H_2N_3)$	146.01	134.05	146.01	134.47
$\Delta(O_4H_5N_6)$	146.01	149.88	146.01	134.47

Table S4. Parameters of bond lengths (Å) and bong angles (Δ°) involved in dual hydrogen bonds for BBTHQ-SSe-PT1, BBTHQ-SSe-PT2 and BBTHQ-SSe-DPT forms in S_0 and S_1 states.

	**BBTHQ-SSe-PT1**		**BBTHQ-SSe-PT2**		**BBTHQ-SSe-DPT**	
	S_0	S_1	S_0	S_1	S_0	S_1
O_1-H_2	0.98914	1.75856	0.98914	1.02572	0.98914	1.75423
H_2-N_3	1.74982	1.03660	1.74982	1.60242	1.74982	1.03609
O_4-H_5	0.98865	1.02229	0.98865	1.77947	0.98865	1.77173
H_5-N_6	1.75384	1.61403	1.75384	1.03409	1.75384	1.03383
$\Delta(O_1H_2N_3)$	146.14	135.66	146.14	150.19	146.14	135.97
$\Delta(O_4H_5N_6)$	146.01	149.79	146.01	134.00	146.01	134.48

Table S5. Vertical excitation energies (λ nm), oscillator strength (*f*), transition compositions and percentages for BBTHQ-SO, BBTHQ-SS and BBTHQ-Se compounds in hexane solvent.

	**Transition**	***λ***	***f***	**Composition**	**CI (%)**
BBTHQ-SO	$S_0 \rightarrow S_1$	433.30	0.646	H → L	98.77
	$S_0 \rightarrow S_2$	346.17	0.881	H-1 → L	96.27
	$S_0 \rightarrow S_3$	320.77	0.098	H-2 → L	94.20
BBTHQ-SS	$S_0 \rightarrow S_1$	446.18	0.641	H → L	98.85
	$S_0 \rightarrow S_2$	354.17	0.836	H-1 → L	96.58
	$S_0 \rightarrow S_3$	325.01	0.001	H-2 → L	94.89
BBTHQ-Se	$S_0 \rightarrow S_1$	450.46	0.649	H → L	98.88
	$S_0 \rightarrow S_2$	361.28	0.594	H-1 → L	96.04
	$S_0 \rightarrow S_3$	339.45	0.027	H-2 → L	94.08

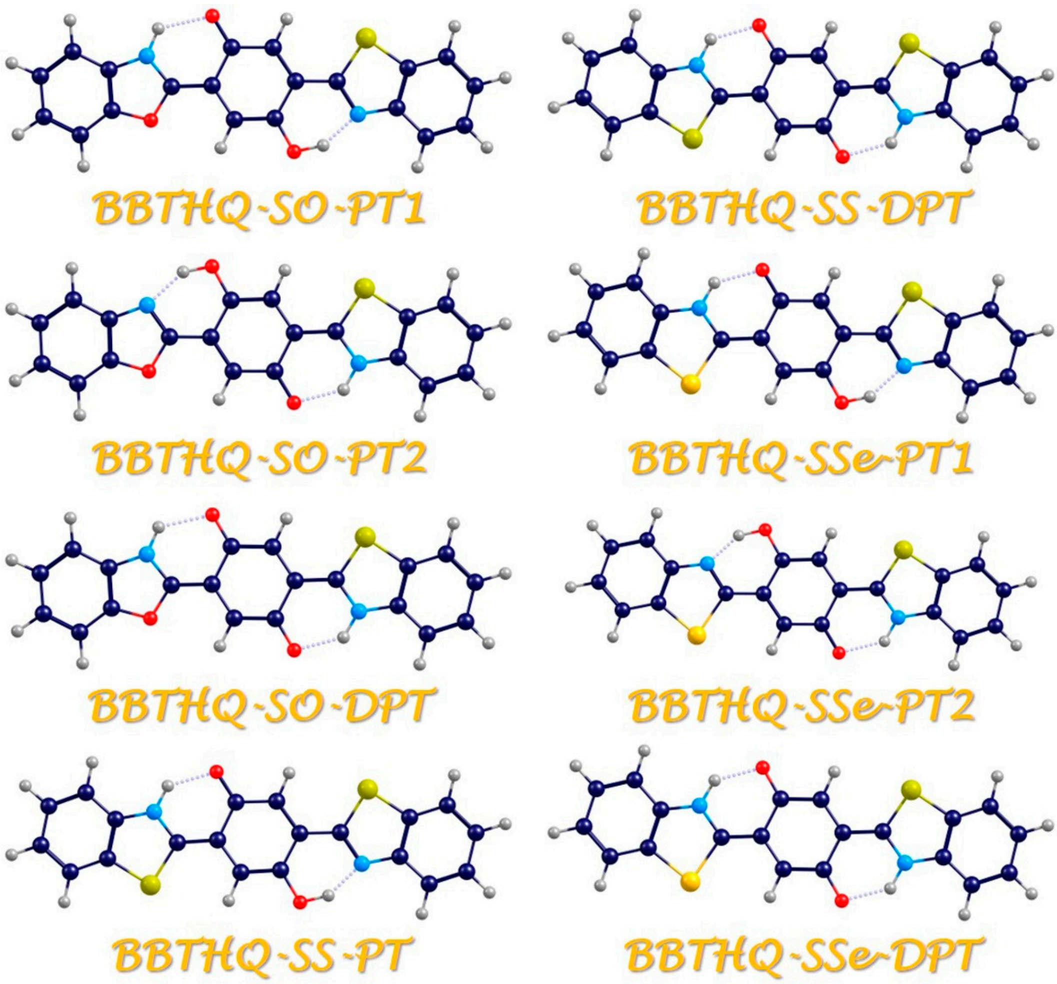

Figure S1. View of the proton-transfer configurations. BBTHQ-SO-PT1: single proton-transfer BBTHQ-SO along with O_1-$H_2\cdots N_3$; BBTHQ-SO-PT2: single proton-transfer BBTHQ-SO along with O_4-$H_5\cdots N_6$; BBTHQ-SO-DPT: double proton-transfer BBTHQ-SO along with O_1-$H_2\cdots N_3$ & O_4-$H_5\cdots N_6$; BBTHQ-SS-PT: single proton-transfer BBTHQ-SS along with O_1-$H_2\cdots N_3$ or O_4-$H_5\cdots N_6$; BBTHQ-SS-DPT: double proton-transfer BBTHQ-SS along with O_1-$H_2\cdots N_3$ & O_4-$H_5\cdots N_6$; BBTHQ-SSe-PT1: single proton-transfer BBTHQ-SSe along with O_1-$H_2\cdots N_3$; BBTHQ-SSe-PT2: single proton-transfer BBTHQ-SSe along with O_4-$H_5\cdots N_6$; BBTHQ-SSe-DPT: double proton-transfer BBTHQ-SSe along with O_1-$H_2\cdots N_3$ & O_4-$H_5\cdots N_6$.

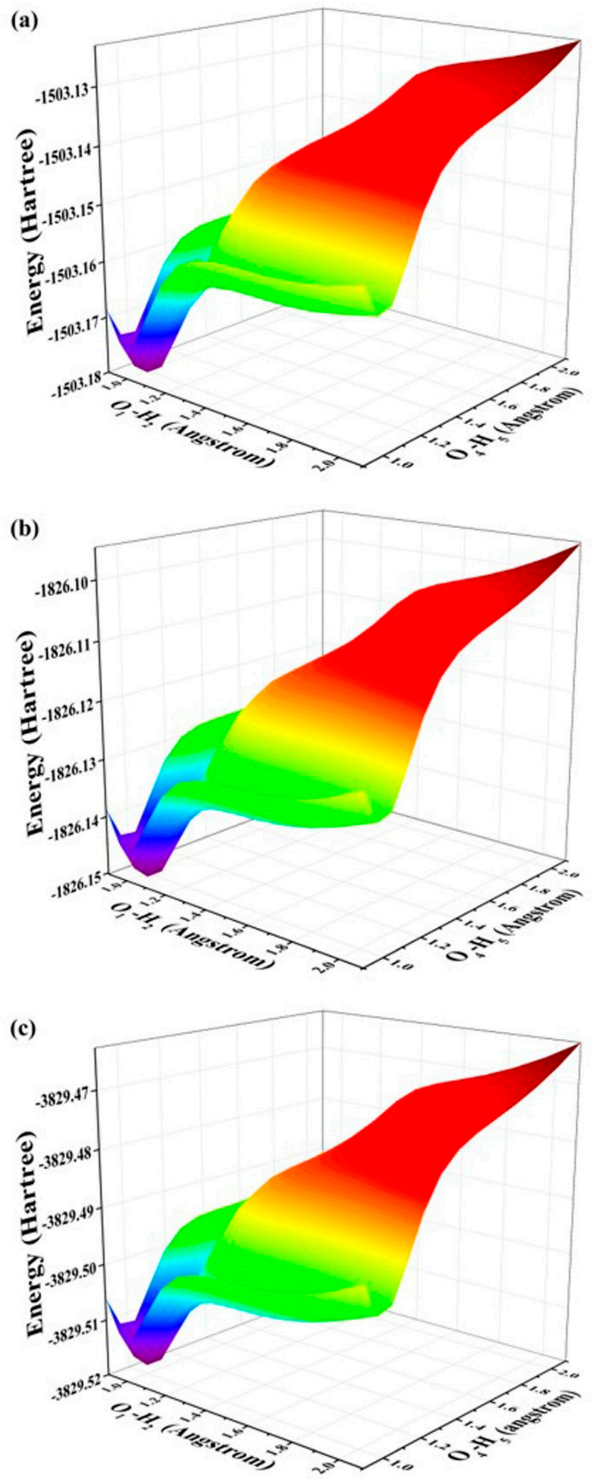

Figure S2. The constructed S_0-sate PES for BBTHQ-SO (a), BBTHQ-SS (b) and BBTHQ-SSe (c).
